# Fluctuations and individual differences in empathy interact with stress to predict mental health, parenting, and relationship outcomes

**DOI:** 10.3389/fpsyg.2023.1237278

**Published:** 2023-10-19

**Authors:** Ido Shalev, Alal Eran, Florina Uzefovsky

**Affiliations:** ^1^Department of Psychology, Ben Gurion University, Beer-Sheva, Israel; ^2^Computational Health Informatics Program, Boston Children's Hospital, Boston, MA, United States

**Keywords:** empathy, stress, depressive symptoms, romantic satisfaction, parenting

## Abstract

**Introduction:**

Empathy is a complex, multifaceted ability allowing for the most basic forms of social communication and plays a prominent role in multiple aspects of everyday lives. In this intensive longitudinal study, we assessed how empathy interacts with stress to predict central domains of psychosocial functioning: mental health, romantic relationships, and parenting.

**Methods:**

Fluctuations and individual differences in empathy were assessed across eight time points, where participants from the general population (*N* = 566) self-reported their empathy, stress, depressive symptoms, romantic satisfaction, and parental functioning.

**Results:**

Both trait and state aspects of empathy were associated with all psychosocial outcomes, with state empathy showing a stronger effect. Additionally, empathy components interacted with stress—emotional empathy better-predicted outcomes under high stress, while cognitive empathy under low stress.

**Discussion:**

Our findings advance the theoretical understanding of empathy, emphasizing the effects of state-dependent empathy fluctuations on our everyday mental and social lives.

## Introduction

Starting from early life, our capacity for empathy is fundamental for our ability to communicate and establish both short-term and enduring social relationships (Uzefovsky and Knafo-Noam, 2016). Empathy significantly influences various aspects of our daily personal and interpersonal interactions (Preston and de Waal, 2003; Decety and Moriguchi, 2007; Decety et al., 2016). Yet, empathy is a complex ability as it consists of multiple components, each context-dependent, showing both trait and state qualities (Davis, 1980; 1983; Zaki, 2014). Our study employs an intensive longitudinal design to comprehensively capture the intricacies of empathy’s components and their interaction with stress, a prominent situational factor, to predict psychosocial functioning in three pivotal domains: mental health, relationships, and parenting. This study was conducted during the onset of the COVID-19 outbreak, which brought about many challenges worldwide (Pillay and Barnes, 2020; Achterberg et al., 2021). Changes such as home-confinement, loneliness, uncertainty about the future, fear for self and others, and loss of loved ones have affected almost all major domains of our daily lives, as well as highlighted individual differences in the stress response to these events (Manchia et al., 2022). Prevalence of psychological problems such as depression and anxiety peaked (Salari et al., 2020; Bueno-Notivol et al., 2021). In the relational aspect, romantic relationships became more unsteady, chaotic and fragile (Goodboy et al., 2021). Families have also been challenged in the parental context with parents reported to be more exhausted (Marchetti et al., 2020). This, along with the involvement of empathy in these three domains (Soenens et al., 2007; Levesque et al., 2014; Bennik et al., 2019), prompted us to focus on depression, relationship satisfaction, and parental capacities as central, albeit not all encompassing, aspects of daily life.

Empathy, a multifaceted ability encompassing cognitive and emotional components, enables us to understand another’s emotions and be affected by them appropriately while retaining self-other differentiation (Decety and Jackson, 2004). Cognitive empathy, also termed perspective-taking or affective Theory of Mind, involves recognizing others’ emotions, whereas emotional empathy entails responding to their mental states with suitable emotions (Baron-Cohen and Wheelwright, 2004). The latter can take two forms: empathic concern occurs when we feel compassion or concern toward the other’s distress, and personal distress occurs when we feel distressed or discomfort in response to the distress of others (Davis, 1980).

Together, the cognitive and emotional aspects of empathy contribute to the most basic forms of social communication (Preston and de Waal, 2003; Decety et al., 2016). As such, empathy plays an indispensable role in interpersonal relationships and has been related to multiple personal and interpersonal outcomes (Wei et al., 2011; Perrone-McGovern et al., 2014; Stern et al., 2015) in ways that are specific to each empathy component (Lamothe et al., 2014; Tone and Tully, 2014; Israelashvili et al., 2020). Our focus centers on three pivotal constructs closely linked to empathy in daily life, each representing a unique aspect of our psychosocial functioning: depressive symptoms, romantic satisfaction, and parenting capacities.

### Depressive symptoms

Depressive symptoms, a widespread and debilitating mental health concern, are typically associated with high levels of personal distress and poor cognitive empathy (Schreiter et al., 2013; Bennik et al., 2019). These alterations in empathy are thought to account for the impaired social function in depression. The association with empathic concern is less clear. For instance, a systematic review found no association between empathic concern and depressive symptoms (Schreiter et al., 2013), yet some studies report that an association between empathic concern and depression emerges in specific personal or situational (e.g., parental support, the identity of the target) contexts (Thomas et al., 2007; Calandri et al., 2019; Salo et al., 2020).

### Relationship satisfaction

Romantic relationships are one of the individuals’ most intimate relationships, and the extent of satisfaction an individual derives from this relationship tends to determine the couple’s stability and overall functioning within the relationship (Fincham and Beach, 2006). Relationship satisfaction was found to be related to all empathy components and is typically associated with higher cognitive empathy and empathic concern, and lower personal distress (Davis and Oathout, 1987; Levesque et al., 2014; Łada and Kaźmierczak, 2019; Cahill et al., 2020).

### Parenting

Parenting encompasses various behaviors and perceptions, relying on numerous cognitive and emotional abilities, such as self-regulation, sensitivity, and self-efficacy (Bornstein et al., 2011; Johnston et al., 2018). Consistently, previous studies suggested that parenting differentially relates to all empathy components: Cognitive empathy and empathic concern predict higher child and parent reports of maternal responsiveness and warmth (Soenens et al., 2007), as well as behavioral measures of maternal sensitivity (Leerkes, 2010). On the other hand, personal distress predicts a higher risk for child abuse, with inconsistent results suggesting a protective role for cognitive empathy and/or empathic concern (Perez-Albeniz and de Paul, 2003, 2004; Meidan and Uzefovsky, 2020).

These associations underscore empathy’s significance across various life domains. However, prior studies primarily examined the trait-level relationship between empathy and these outcomes, even though empathy might fluctuate and be affected by situational factors (Zaki, 2014; Fabi et al., 2019; Depow et al., 2021). One substantial contextual factor, stress, is of notable importance (Diehl et al., 2012). Stress elicits self-protective reactions (like fight, flight, or freeze) as well as prompts supportive and caregiving behaviors (termed tend and befriend; Taylor, 2006). Stress has been directly associated with both mental-health and interpersonal relationship outcomes (Yang et al., 2015; Crnic and Ross, 2017; Randall and Bodenmann, 2017). However, given empathy’s role in nurturing care (Stern and Cassidy, 2018), it is also likely that stress could modify the interplay between empathy components and the diverse psychosocial outcomes related to it. For example, one might intuitively suggest that the ability to feel concerned for others in need (i.e., empathic concern) might be related more strongly to parents’ perceptions and behaviors under stressful conditions, which trigger tend and befriend responses. Indeed, cognitive and emotional empathy were found to interact with stress to predict psychosocial functioning such as aggressive parenting, decision-making, and psychological growth after experiencing traumatic events (Letourneau, 1981; Zhang et al., 2019; Hu et al., 2021). These studies focused on empathy as a resilience factor, whereby higher empathy was found to ameliorate the harmful effects of stress. Yet the relationship between stress and empathy may be more complex than that (Tone and Tully, 2014). Moreover, the dynamic nature of empathic feelings was not taken into account, and this is the focus of the current study.

Beyond variation between individuals, empathy can be prompted (Klimecki et al., 2016; Shaffer et al., 2019) and can also fluctuate intrinsically within the same person (Zaki, 2014; Fabi et al., 2019; Depow et al., 2021), showing both between- and within-person variability. Although research on empathy’s fluctuations hints at their distinct implications compared to trait empathic capacities (Davis, 1983; Fabi et al., 2019), these studies are relatively scarce and often concentrates on responses to a specific stimuli, sometimes overlooking individual differences (Hein et al., 2018; Jauniaux et al., 2021). Consequently, a comprehensive approach considering both fluctuations and between-person differences in empathy is pivotal for unraveling the intricate relationships among empathy, stress, and psychosocial functioning.

#### The current study

This study aimed to evaluate the associations between fluctuations and individual differences in empathy, and key psychosocial functioning: depressive symptoms, relationship satisfaction, and parenting. In this study, participants completed empathy assessments and related measures at eight intervals spanning three months, coinciding with the onset of the COVID-19 outbreak. Throughout this period, Israel witnessed its first lockdown and substantial restrictions, marked by heightened uncertainty, profound alterations to personal surroundings, and elevated stress levels (Planchuelo-Gómez et al., 2020; Achterberg et al., 2021). The turbulent and precarious nature of the COVID-19 pandemic resulted in pronounced fluctuations in stress levels, allowing us to examine the intricate dynamics between empathy fluctuations, stress, and their relationships with psychosocial outcomes.

Based on the reviewed literature, we hypothesized regarding the individual differences in the empathy components. We hypothesized that favorable outcomes (e.g., parenting capacities, romantic satisfaction) would correlate with heightened cognitive empathy and/or empathic concern, as well as with reduced personal distress. Conversely, negative outcomes (e.g., depression) were hypothesized to manifest opposite pattern. Additionally, we hypothesized that both parenting and relationship satisfaction would be associated with all three components of empathy (Perez-Albeniz and de Paul, 2003, 2004; Soenens et al., 2007; Levesque et al., 2014; Righetti et al., 2016; Łada and Kaźmierczak, 2019; Meidan and Uzefovsky, 2020). Furthermore, we expected that depression would exhibit a stronger association with individual variations in cognitive empathy and personal distress, while demonstrating a relatively weaker association with empathic concern (Schreiter et al., 2013; Domes et al., 2016; Bennik et al., 2019).

We further extended our hypotheses to encompass within-individual empathy fluctuations. Due to the scarcity of preceding literature investigating such fluctuations, our hypotheses held a less firm foundation. Drawing from Taylor’s evolutionary theory (2006), we hypothesized that stress would serve as a modulating factor influencing empathy’s interaction with psychosocial functioning, particularly becoming more dominant during elevated stress levels. That is, we expected that the associations described above would be stronger when stress levels were higher.

## Materials and methods

This study was approved by the Human Subject Committee of Ben-Gurion University. All participants gave informed consent before participation. This study was not formally pre-registered.

### Power analysis

A Monte-Carlo power analysis with 5,000 simulations (as recommended by Mundfrom et al., 2011) indicated that 284 participants are sufficient (power greater than 0.8) to detect small to medium effect sizes, even with a strict Bonferroni-corrected *p*-value of 0.01. The power analysis was conducting using the “simr” package v1.0.5 (Green and MacLeod, 2016).

### Procedure, participants, and data preprocessing

This longitudinal study was designed to capture changes in empathy and psychosocial functioning during the onset of the first wave of the COVID-19 pandemic in Israel (mid-March to mid-May, 2020). The pandemic led to restrictions, including social distancing, and a first full lockdown imposed by the end of March. Restrictions gradually lifted in mid-April and continued until May 19th (Last, 2020).

Participants completed a battery of online questionnaires followed by weekly reports at eight different time points measuring their levels of empathy, stress, depression, relationship satisfaction, and parental functioning. The first six weekly assessments were collected starting one week after the lockdown was imposed (April 1st to May 8th, 2020), and additional two biweekly assessments were collected after the restrictions were lifted (May 21st to June 7th, 2020).

We anticipated attrition to be very high due to the longitudinal design of this study as well as the uncertainty involved at the onset of a global pandemic. Therefore, we decided to recruit twice the number of participants suggested by the power analysis. To do so, participants were recruited using two strategies: (a) *N* = 99 participants were undergraduate psychology students from Ben-Gurion University are typically rewarded with additional 5 points to their final grade in an introductory class if they participate in a set number of research hours; (b) *N* = 467 participants were parents (not couples) of 3–10 year-old children (mean 5.02 ± 3.71) recruited via Ha’midgam, a survey company conducting online surveys in Israel. If a parent had more than one child, they were directed to report on the youngest child who is older than three years old. The two samples were intended to provide more comprehensive data by encompassing a wider age range and different stages of adulthood (e.g., the students in our sample had no children). Relationship satisfaction was completed only for participants who were currently in a committed relationship (*N* = 498). Additionally, students in Israel tend to be older than in other parts of the world (Mean age of the students’ sample = 23.70 ± 3.03) and therefore are more likely to engage in long-term committed relationships. Overall, 566 individuals participated in the first time-point.

To ensure participants were paying attention throughout the study, we randomly included attention checks (e.g., “sometimes people do not read all the items. If you read this, please mark 4”) for all key questionnaires. If a participant did not pass an attention check for a specific measure, that specific response was removed from the specific timepoint.

Participants were excluded from a specific timepoint if they failed two or more attention checks or if they left more than half of all the questions unanswered. After exclusion, the first time-point included 546 participants (77% females, mean age = 31.8 ± 6.52). Supplementary Table S1 summarizes the total number of participants excluded at each time-point.

Questionnaire responses with over 10% missing items were considered missing at construct-level and were removed. No outliers were removed. Overall, in this study 117 unique responses were removed leaving 3,014 unique responses across the entire study period. Missing data were imputed using the “mice” package v3.13.0 in R (Buuren and Groothuis-Oudshoorn, 2010). Descriptive statistics and the total number of participants analyzed at each time point are summarized in Supplementary Table S2.

### Measures

Validated Hebrew versions were used for measuring depression (Shmotkin and Keinan, 2011). The empathy measure was adapted from a version previously used elsewhere (Uzefovsky et al., 2014; Meidan and Uzefovsky, 2020). All other measures were translated into Hebrew and back-translated to verify the appropriateness of the translation. In addition to the measures described below, we also measured the Childhood Trauma Questionnaire (Bernstein et al., 1994) as part of another unrelated study conducted in the lab. We reported all manipulations, measures, and exclusions in this study.

### Empathy

Empathy was measured using the Interpersonal Reactivity Index (IRI) (Davis, 1980). The IRI is a self-report questionnaire that consists of four validated subscales, each made up of seven items. Two subscales tap emotional aspects of empathy. The “empathic concern” subscale assesses feelings of compassion and concern toward the other, while “personal distress” assesses the tendency to experience distress or discomfort in response to other’s distress. The other two subscales tap into the cognitive aspects of empathy. The “perspective-taking” subscale measures the tendency to adopt the psychological view of others, while the “fantasy” subscale assesses the tendency to imaginatively transpose oneself into the feelings and actions of fictional characters. As the “fantasy” scale is considered more controversial and harder to interpret (Davis, 1994; De Corte et al., 2007), we excluded this subscale, leaving 21 items scored on a 5-point scale. The IRI was adapted for weekly measurements asking the participants to report how well each statement described them during the last week and urged them to imagine how they would have reacted to the mentioned situation if that occurred during the preceding week. Cronbach’s α of the subscales across all time points were 0.78 for empathic concern, 0.69 for personal distress, and 0.85 for perspective-taking.

### Stress

Current stress was measured using a single-item stress measure with satisfactory psychometric properties (Elo et al., 2003). Participants were presented with the following definition of stress: “Stress means a situation in which a person feels tense, restless, nervous or anxious, or is unable to sleep at night because his/her mind is troubled all the time. Do you feel this kind of stress these days?.” This item was rated on a 5-point scale from 1 (“not at all”) to 5 (“very much”).

### Depressive symptoms

Depressive symptoms were assessed weekly using the 5-item version of the Center for Epidemiological Studies Depression Scale (CES-D) (Shrout and Yager, 1989; Bohannon et al., 2003). Participants reported their depressive symptoms in the last week on a 4-point scale ranging from 0 (“rarely or none of the time”) to 3 (“most or all the time”). Scores ranged from 0–15, with a higher score indicating higher severity of symptoms. A cut-off of ≥5.5 was used for assessing clinically significant symptoms (Bohannon et al., 2003). Cronbach’s α for this measure across all time points was 0.83.

### Parenting

We assessed different aspects of parental capacities during the lockdown. To do so, we used measures of parenting functioning adapted for weekly measurement that was comprised of items from two questionnaires. The first measure was derived from the Alabama Parenting Questionnaire (APQ) (Frick, 1991). We selected three items (“you let your child know when he/she is doing a good job with something,” “you compliment your child after he/she has done something well,” and “you praise your child if he/she behaves well”) from the “positive parenting” subscale that assesses the parent’s use of positive reinforcement.

The second questionnaire used was the Parental Burnout Inventory (PBI) (Roskam et al., 2017). Two subscales were used from this inventory; the “emotional exhaustion” subscale assessing exhaustion in one’s parental role (four items) and the “parental accomplishment” subscale that measures parental efficacy and accomplishment (six items) (for a description of all items used, see Supplementary Table S3).

The APQ and PBI typically assess general/yearly frequency of behavior with a scale ranging from “never” to “always/every-day” on a 5/7-items scale (respectively). We adapted these for weekly measurements using a 4-point response scale: 1 = “rarely or never (less than one day),” 2 = “some or small part of the time (1–2 days),” 3 = “sometimes or often (3–4 days),” and 4 = “most or all the time (5–7 days).” Participants rated how often they felt or behaved as described in the statement over the past week. To make sure the derived items still preserved the meaningful structure of the original three factors, we used exploratory factor analysis on the 13 items (scree plot and factor loadings of the factor analysis are shown in Supplementary Figure S1 and Supplementary Table S3). This analysis revealed three optimal factors entirely identical to the three subscales used—“positive parenting,” “parental accomplishment,” and “emotional exhaustion.” Factor analysis was conducted using the “psych” package v2.1.6 in R (Revelle, 2017). Cronbach’s α of each subscale across all time points were 0.81 for “emotional exhaustion,” 0.91 for “parental accomplishment,” and 0.77 for “positive parenting.”

### Relationship satisfaction

Relationship satisfaction was measured (if participants were currently in a relationship) using the 4-item version of the Couple Satisfaction Index (CSI-4) (Funk and Rogge, 2007). The CSI-4 measures individuals’ global evaluation of their romantic relationships. The first item is rated on a 7-point scale from 0 (“extremely unhappy”) to 6 (“perfect”), and the other three items on a 6-point scale from 0 (“not at all”) to 5 (“completely”). We instructed the participants to answer these items relating to the last week. Scores are then summed with a higher score representing higher relationship satisfaction. Cronbach’s α for this measure across all time points was 0.96.

### Statistical analysis

As all measures were self-reported by the participants, we assessed the common method bias, using a Harman’s one-factor test (Podsakoff and Organ, 1986). An unrotated exploratory factor analysis that included the key self-reports measures used in this study identified five factors with eigenvalues greater than 1. The first factor accounted for only 17.90% of the total variance, falling below the 50% threshold. Therefore, the common method bias is unlikely to substantially affect the results of this study.

To account for the longitudinal structure of our data, we performed a two-level multilevel modeling (MLM) where multiple observations at the different time-points were nested within participant. As empathy and its components typically differ by sex (Derntl et al., 2010), and due to the variability in age in the current sample, we first made sure that age and sex were accounted for in all analyses. To distinguish between within-person (fluctuations) and between-person (individual differences) variances we used two different methods of centering (Raudenbush and Bryk, 2002). For each IRI subscale (perspective taking, empathic concern, and personal distress), observations were centered based on the person-mean, representing within-person deviations of the participant from their own mean (i.e., state empathy). Then, person-level empathy score (the mean of the person across all points) was centered on the grand mean representing how the mean of each participant across all time points (i.e., trait empathy) deviated from the mean of the sample (i.e., between-person variance). Finally, stress was included in the models, and we inspected its role as a moderator for the association between empathy and the outcome (list of models compared are summarized in Supplementary Table S4). Interaction with stress was probed as a continuous variable. Further analyses were probed by stratifying stress into three-levels—“Low stress” includes ratings of 1 (“not at all”) and 2 (“only a little”), “Medium stress” includes a rating of 3 (“to some extent”); and “High stress” includes ratings of 4 (“rather much”) and 5 (“very much”).

These same analyses were performed for each outcome separately (depressive symptoms, relationship satisfaction, and the three parenting measures), except that in the depression and relationship satisfaction models, we also controlled for differences between samples (the students’ sample did not include parents and was therefore not included in those analyses). To account for multiple testing, we assigned a strict Bonferroni-corrected *p*-value of *p* < 0.01.

All analyses were carried out using R v4.0.3 (R Core Team, 2014). Multilevel model analyses were conducted using the “nlme” package v3.1–152 (Pinheiro and Bates, 2021). Interactions were probed using the “interactions” package v1.1.15 (Long, 2019).

## Results

Table 1 shows a correlation matrix of bivariate Pearson’s correlations, using subject as a random variable.

**Table 1:** Bivariate Pearson’s correlation across time.

	1	2	3	4	5	6	7	8
1. IRI—empathic concern	–							
2. IRI—personal distress	0.02	–						
3. IRI—perspective-taking	0.44***	−0.13***	–					
4. Stress	−0.01	0.31***	−0.06***	–				
5. CES-D	−0.05**	0.37***	−0.08***	0.57***	–			
6. APQ—positive parenting	0.14***	−0.1***	0.12***	−0.05*	−0.12***	–		
7. PBI—emotional exhaustion	−0.03	0.32***	−0.1***	0.16***	0.45***	−0.19***	–	
8. PBI—parental accomplishment	0.14***	−0.24***	0.14***	−0.18***	−0.18***	0.45***	−0.36***	–
9. CSI	0.13***	−0.21***	0.17***	−0.23**	−0.35***	0.21***	−0.27***	0.32***

IRI, Interpersonal Reactivity Index; CES-D, Center for Epidemiological Studies Depression Scale; APQ, Alabama Parenting Questionnaire; PBI, Parental Burnout Inventory; CSI, Couple Satisfaction Index. *p < 0.05, **p < 0.005, ***p < 0.0005.

Only a minority of the participants reported that they were at risk for developing complications of COVID-19 (9%), with only a few participants diagnosed throughout the study (see Supplementary Table S2). This is in line with the relatively low rates of COVID-19 infection in Israel reported at that time.

Importantly, 36.5% of individuals exceeded the cut-off of the CES-D (Bohannon et al., 2003) for at least one time-point. This high rate of depressive symptoms is consistent with other studies showing that the COVID-19 pandemic increased depression in the general population in Israel and worldwide (Amit Aharon et al., 2021; Bueno-Notivol et al., 2021).

We also examined whether individuals who participated in less than 50% of the time points differed in their reported income, stress, or depression levels from participants who participated in most, or all, of the study. The two groups of participants did not differ in income [*t*(544) = −0.51, *p* = 0.61], stress [*t*(2981) = 0.23, *p* = 0.82], nor depression levels [*t*(2974) = −0.52, *p* = 0.60], suggesting attrition was unrelated to these characteristics and might be at random. Descriptive statistics of empathy components, stress, and outcome measures are summarized in Supplementary Table S5.

### Intraclass correlation of IRI

The intraclass correlation for the empathy components was 73.1% for empathic concern, 66.8% for personal distress, and 76.5% for perspective-taking, reflecting that most of the variance in empathy was due to individual differences, while 23.5–33.2% of the variance in empathy components throughout the lockdown was due to within-person fluctuations.

Below, each outcome is discussed separately, with Tables 1–5 depicting the findings of each final model. For a full comparison of the models used in the MLM analyses (as listed in Supplementary Table S4) and their variance-covariances matrices, see Supplementary Tables S6, S7, respectively. Residuals for all models were approximately normally distributed.

### Depressive symptoms

Results for the depressive symptoms as the outcome are reported in Table 2.

**Table 2:** Multilevel model with depressive symptoms as the outcome.

Parameter		Estimate	95% CI	*p*-value	Standardized estimate (*β*)
	Intercept	−0.5	[−1.69, 0.68]	0.41	
	Sex	0.24	[−0.11, 0.6]	0.19	0.04
	Age	0.01	[−0.02, 0.03]	0.68	0.02
	Sample***	**1.08**	**[0.61, 1.56]**	**0.00001**	**0.15**
	Stress***	**1.04**	**[0.96, 1.12]**	**1 × 10** ^**−124**^	**0.38**
Individual differences	Empathic concern	−0.07	[−0.16, 0.02]	0.13	−0.1
	Personal distress	0.03	[−0.05, 0.1]	0.45	0.04
	Perspective-taking	−0.02	[−0.09, 0.06]	0.69	−0.03
	Empathic concern × stress	0.02	[−0.01, 0.05]	0.18	0.07
	**Personal distress** ×stress***	**0.1**	**[0.07, 0.12]**	**1×10** ^**−16**^	**0.36**
	Perspective-taking × stress	−0.002	[−0.03, 0.02]	0.85	−0.01
Fluctuations	**Empathic concern** ^**†**^	**0.08**	**[0.003, 0.15]**	**0.04**	**0.09**
	Personal distress	0.05	[−0.01, 0.11]	0.12	0.06
	**Perspective-taking** ^**†**^	**−0.09**	**[−0.16, −0.02]**	**0.01**	**−0.11**
	Empathic concern × stress**	**−0.05**	**[−0.08, −0.02]**	**0.0003**	**−0.15**
	Personal distress × stress*	**0.04**	**[0.01, 0.06]**	**0.004**	**0.11**
	**Perspective-taking × stress** ^†^	0.03	[0.003, 0.06]	0.03	0.1
	**Marginal *R*** ^**2**^ **/Conditional *R*** ^**2**^	**0.46/0.72**			

^†^*p* < 0.05, *p < 0.01, **p < 0.001, ***p < 0.0001. Significant effects are denoted in bold.

#### Empathic concern

**Figure 1:**
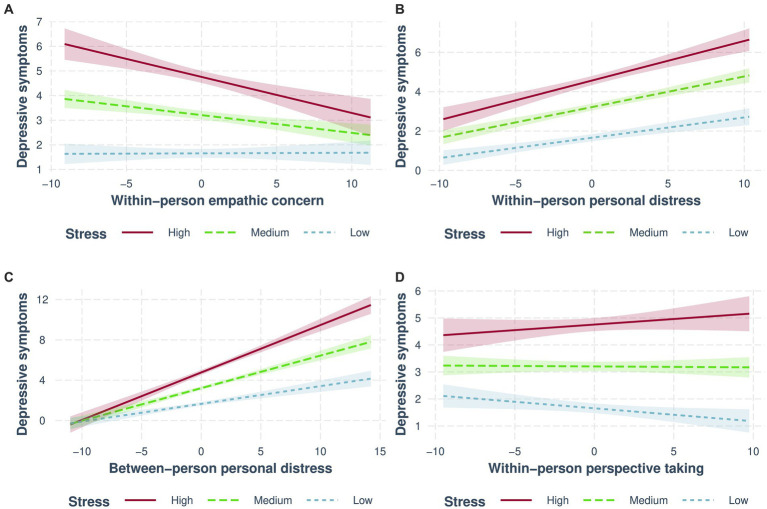
Interactions between empathy and stress predict depressive symptoms. More solid lines colored blue, green, and red (respectively) represent low, medium, and high levels of stress. Different plots are displayed for **(A)**. Within-person empathic concern; **(B)** within-person personal distress; **(C)** between-person personal distress; and **(D)** within-person perspective-taking.

Fluctuations in empathic concern throughout the lockdown interacted with stress to predict depressive symptoms. Examining this interaction (see Figure 1A) revealed that changes in empathic concern were negatively associated with depressive symptoms when stress levels were medium (*b* = −0.07, 95% CI, [−0.11, −0.04], *β* = −0.08, *p* = 0.00005) or high (*b* = −0.15, 95% CI [−0.21, −0.08], *β* = −0.17, *p* = 0.00001), but were not associated with depressive symptoms when stress levels were low (*b* = 0.002, 95% CI [−0.04, 0.04], *β* = 0.003, *p* = 0.91). The effect of the individual differences in empathic concern was non-significant.

#### Personal distress

Fluctuations in personal distress interacted with stress to predict depressive symptoms (see Figure 1B), such that the association between depressive symptoms and fluctuations in personal distress became stronger when stress increased from low (*b* = 0.10 95% CI, [0.07, 0.14], *β* = 0.18, *p* = 1 × 10^−8^) to medium (*b* = 0.16, 95% CI [0.12, 0.19], *β* = 0.19, *p* = 1×10^−21^), to high (*b* = 0.21, 95% CI [0.15, 0.27], *β* = 0.25, *p* = 2 × 10^−12^).

The same patterns were found for individual differences in personal distress (see Figure 1C) showing a stronger positive association between personal distress and depressive symptoms as the level of stress increased from low (*b* = 0.18, 95% CI [0.12, 0.22], *β* = 0.36, *p* = 3 × 10^−11^) to medium (*b* = 0.32, 95% CI [0.28, 0.37], *β* = 0.47, *p* = 9 × 10^−37^), to high (*b* = 0.47, 95% CI [0.41, 0.53], *β* = 0.54, *p* = 1 × 10^−43^).

#### Perspective-taking

Although nominally significant and did not pass a multiple testing correction, fluctuations in perspective-taking were associated with lower depressive symptoms. However, this result was qualified by an interaction with stress (see Figure 1D), as perspective-taking was nominally and negatively associated with depressive symptoms only when stress levels were low (*b* = −0.05, 95% CI [−0.09, −0.01], *β* = −0.08, *p* = 0.02). The simple slopes were non-significant for medium (*b* = −0.004, 95% CI [−0.04, 0.03], *β* = −0.004, *p* = 0.84) and high (*b* = 0.04, 95% CI [−0.02, 0.1], *β* = 0.05, *p* = 0.18) levels of stress. The effect of between-person perspective-taking was non-significant.

### Relationship satisfaction

Results for relationship satisfaction as the outcome are reported in Table 3.

**Table 3:** Multilevel model with relationship satisfaction as the outcome.

	Parameter	Estimate	95% CI	*p*-value	Standardized estimate (*β*)
	Intercept***	**25.29**	**[22.29, 28.29]**	**3×10** ^**−48**^	
	Sex	0.55	[−0.39, 1.48]	0.26	0.05
	Age***	**−0.18**	**[−0.25, −0.11]**	**0.000001**	**−0.25**
	Sample	−0.79	[−2.17, 0.59]	0.26	−0.05
	Stress***	**−0.4**	**[−0.54, −0.26]**	**5 × 10** ^**−8**^	**−0.09**
Individual differences	Empathic concern	0.14	[−0.06, 0.33]	0.17	0.11
	Personal distress*	**−0.25**	**[−0.4, −0.09]**	**0.002**	**−0.19**
	Perspective-taking	0.12	[−0.04, 0.29]	0.15	0.11
	Empathic concern × stress	−0.04	[−0.09, 0.01]	0.13	−0.08
	**Personal distress × stress** ^**†**^	**−0.05**	**[−0.09, −0.01]**	**0.02**	**−0.11**
	Perspective-taking × stress	0.01	[−0.03, 0.05]	0.62	0.03
Fluctuations	Empathic concern	−0.03	[−0.15, 0.09]	0.65	−0.02
	Personal distress*	**−0.17**	**[−0.27, −0.06]**	**0.002**	**−0.15**
	Perspective-taking	0.08	[−0.04, 0.2]	0.17	0.07
	Empathic concern × stress	0.04	[−0.002, 0.09]	0.06	0.09
	Personal distress × stress	0.02	[−0.02, 0.06]	0.27	0.05
	Perspective-taking × stress	0.02	[−0.03, 0.06]	0.43	0.04
	**Marginal *R*** ^**2**^ **/Conditional *R*** ^**2**^	**0.18/0.77**			

^†^*p* < 0.05, *p < 0.01, **p < 0.001, ***p < 0.0001. Significant effects are denoted in bold.

**Figure 2:**
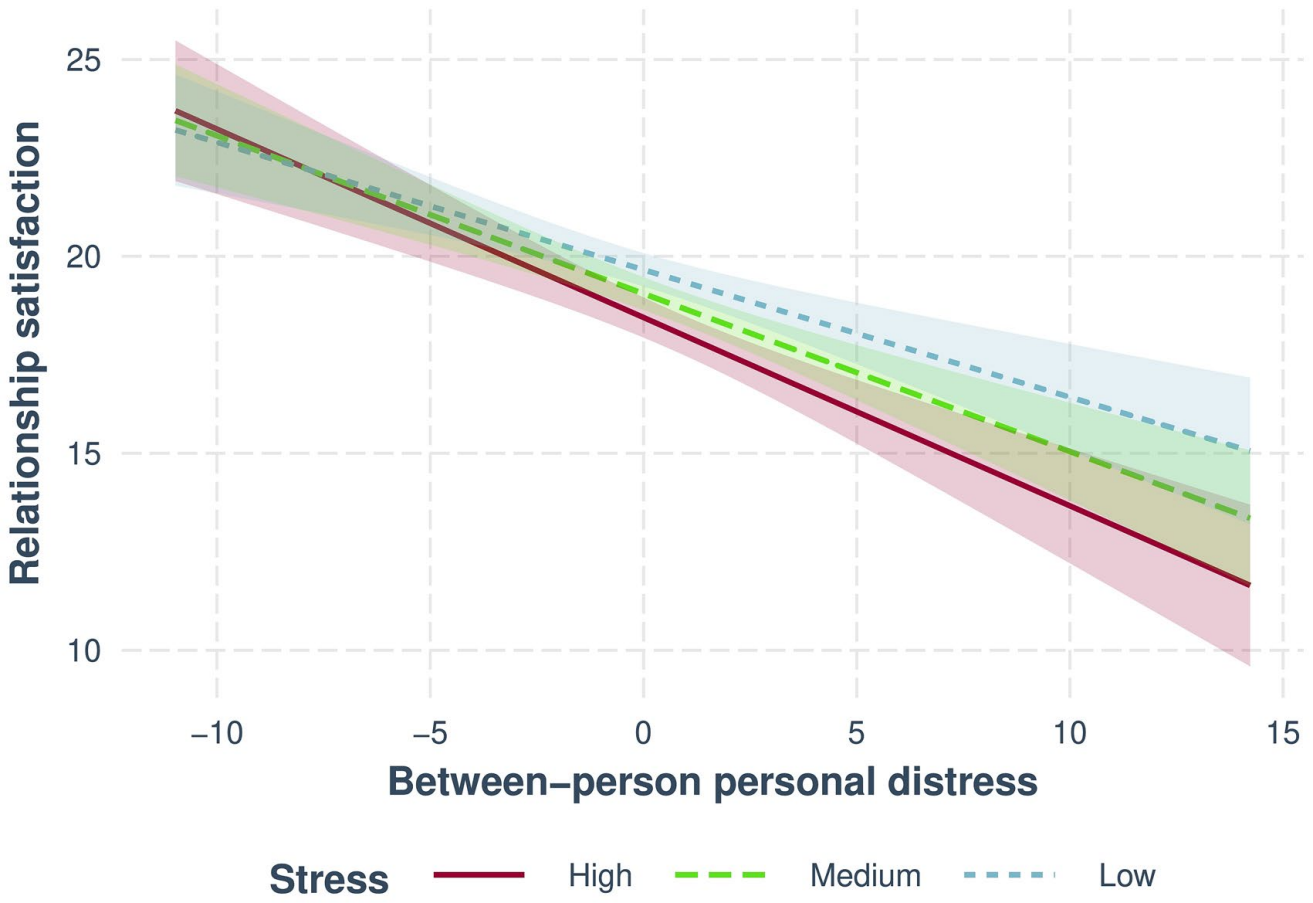
Interaction between between-person personal distress and stress predicts relationship satisfaction. More solid lines colored blue, green, and red (respectively) represent low, medium, and high levels of stress.

Only personal distress was negatively associated with relationship satisfaction at both individual differences and fluctuations. Individual differences in personal distress also interacted with stress (see Figure 2), with a stronger association found between personal distress and relationship satisfaction as the level of stress increases from low (*b* = −0.32, 95% CI [−0.45, −0.2], *β* = −0.24, *p* = 0.000001) to medium (*b* = −0.4, 95% CI [−0.52, −0.28], *β* = −0.24, *p* = 2 × 10^−10^), to high (*b* = −0.48, 95% CI [−0.63, −0.33], *β* = −0.28, *p* = 3 × 10^−10^) levels of stress.

### Parenting

**Figure 3:**
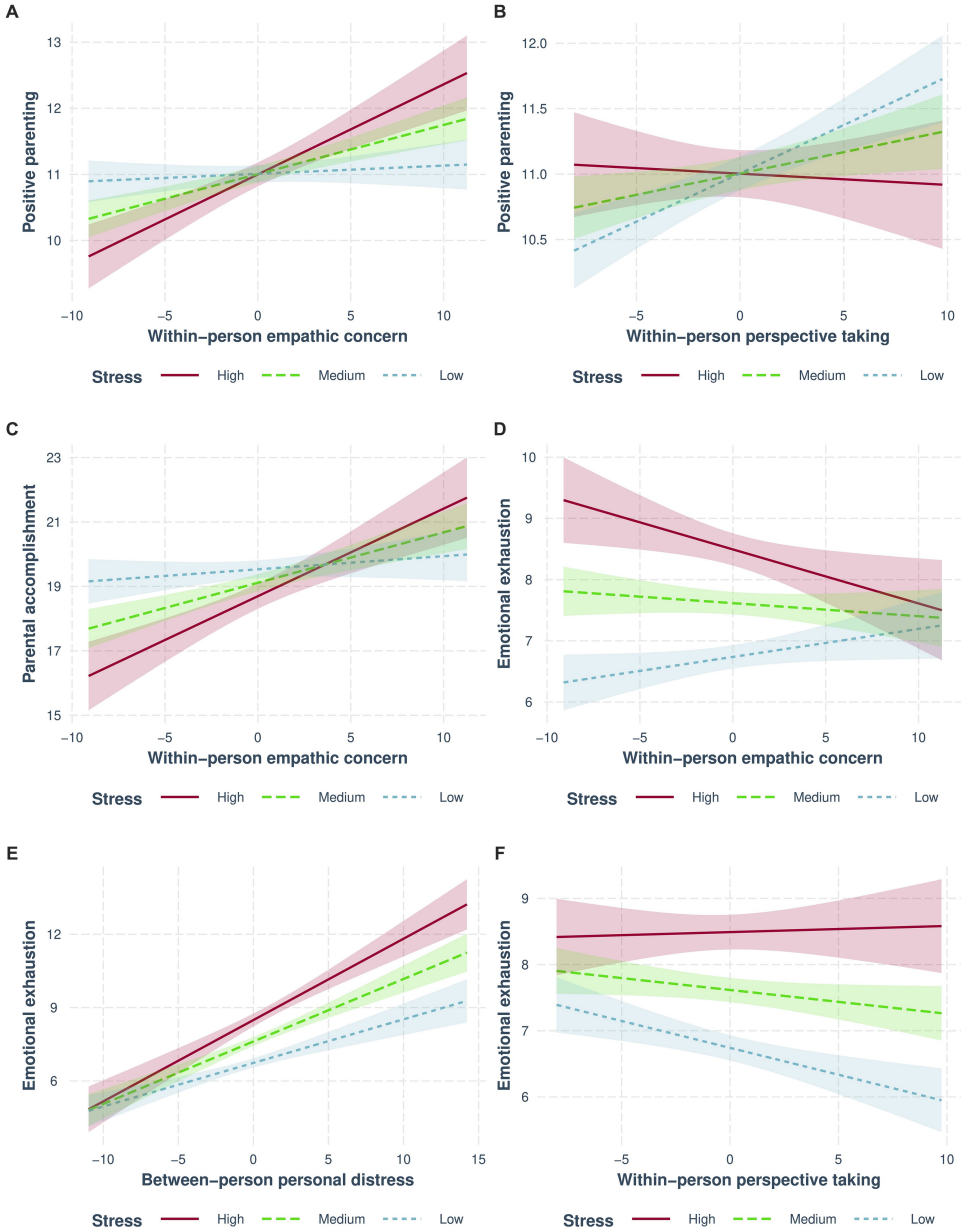
Interactions between empathy and stress predict parental functioning. More solid lines colored blue, green, and red (respectively) represent low, medium, and high levels of stress. Different plots are displayed for “positive parenting” **(A,B)**, “parental accomplishment” **(C)**, and “emotional exhaustion” **(D–F)** subscales. Within-person empathic concern interacted with stress to predict all parental capacities **(A,C,D)**. **(B,F)** Show the interaction between within-person perspective-taking and stress, while the interaction between stress and between-person personal distress is shown in **(E)**.

Results for parenting are reported in Table 4 for positive parenting (Table 4A), parental accomplishment (Table 4B), and emotional exhaustion (Table 4C). The interaction effects are presented in Figure 3.

**Table 4:** Multilevel model with positive parenting (A), parental accomplishment (B), and emotional exhaustion (C) as the outcomes.

	Parameter	Estimate	95% CI	*p*-value	Standardized estimate (*β*)
(A) Positive parenting					
	Intercept***	**11.48**	**[10.56, 12.4]**	**6 × 10** ^**−84**^	
	Sex***	**0.66**	**[0.38, 0.94]**	**0.00001**	**0.23**
	Age***	**−0.05**	**[−0.07, −0.03]**	**0.00001**	**−0.21**
	Stress	−0.004	[−0.07, 0.06]	0.9	−0.003
Individual differences	**Empathic concern** ^**†**^	**0.07**	**[0.001, 0.14]**	**0.05**	**0.2**
	Personal distress	−0.05	[−0.11, 0.01]	0.08	−0.14
	Perspective-taking	0.03	[−0.03, 0.09]	0.33	0.1
	Empathic concern × stress	0.02	[−0.004, 0.04]	0.12	0.13
	Personal distress × stress	−0.02	[−0.03, 0.001]	0.07	−0.12
	Perspective-taking × stress	−0.01	[−0.03, 0.01]	0.28	−0.09
Fluctuations	Empathic concern	−0.05	[−0.1, 0.01]	0.08	−0.09
	Personal distress	−0.04	[−0.08, 0.01]	0.15	−0.07
	Perspective-taking***	**0.12**	**[0.06, 0.17]**	**0.0001**	**0.21**
	Empathic concern × stress***	**0.04**	**[0.02, 0.06]**	**0.0001**	**0.21**
	Personal distress × stress	−0.003	[−0.02, 0.02]	0.78	−0.01
	Perspective-taking × stress*	**−0.03**	**[−0.05, −0.01]**	**0.01**	**−0.14**
	**Marginal *R*** ^**2**^ **/Conditional *R*** ^**2**^	**0.19/0.59**			
(B) Parental accomplishment					
	Intercept***	**21.91**	**[19.97, 23.84]**	**1×10** ^**−72**^	
	Sex	0.59	[−0.001, 1.18]	0.05	0.09
	Age**	**−0.09**	**[−0.13, −0.04]**	**0.0001**	**−0.17**
	Stress***	**−0.28**	**[−0.42, −0.14]**	**0.0001**	**−0.08**
Individual differences	Empathic concern**	**0.29**	**[0.14, 0.44]**	**0.0002**	**0.36**
	Personal distress***	**−0.31**	**[−0.43, −0.19]**	**0.000001**	**−0.37**
	Perspective-taking	0.08	[−0.05, 0.22]	0.2	0.12
	Empathic concern × stress	−0.03	[−0.07, 0.02]	0.29	−0.08
	Personal distress × stress	0.001	[−0.04, 0.04]	0.95	0.003
	Perspective-taking × stress	0.005	[−0.04, 0.05]	0.8	0.02
Fluctuations	Empathic concern	−0.07	[−0.2, 0.05]	0.24	−0.06
	Personal distress*	**−0.17**	**[−0.27, −0.06]**	**0.002**	**−0.15**
	Perspective-taking**	**0.21**	**[0.09, 0.34]**	**0.001**	**0.17**
	Empathic concern × stress***	**0.08**	**[0.03, 0.12]**	**0.001**	**0.17**
	Personal distress × stress	−0.02	[−0.06, 0.02]	0.26	−0.05
	Perspective-taking × stress	−0.04	[−0.09, 0.001]	0.06	−0.1
	**Marginal *R*** ^**2**^ **/Conditional *R*** ^**2**^	0.27/0.61			
(C) Emotional exhaustion					
	Intercept***	**5.72**	**[4.37, 7.05]**	**1×10** ^**−15**^	
	**Sex** ^**†**^	**−0.47**	**[−0.87, −0.06]**	**0.03**	**−0.1**
	Age	0.03	[−0.002, 0.06]	0.07	0.08
	Stress***	**0.58**	**[0.49, 0.67]**	**2×10** ^**−34**^	**0.25**
Individual differences	Empathic concern	0.01	[−0.09, 0.11]	0.81	0.02
	**Personal distress** ^**†**^	**0.1**	**[0.02, 0.18]**	**0.02**	**0.17**
	Perspective-taking	−0.03	[−0.12, 0.06]	0.48	−0.06
	Empathic concern × stress	−0.01	[−0.05, 0.02]	0.37	−0.07
	Personal distress × stress**	**0.05**	**[0.03, 0.08]**	**0.0001**	**0.23**
	Perspective-taking × stress	−0.02	[−0.04, 0.01]	0.22	−0.09
Fluctuations	Empathic concern*	**0.11**	**[0.03, 0.19]**	**0.01**	**0.13**
	Personal distress***	**0.17**	**[0.1, 0.24]**	**0.000001**	**0.23**
	Perspective-taking*	**−0.13**	**[−0.21, 0.05]**	**0.002**	**−0.15**
	Empathic concern × stress*	**−0.05**	**[−0.07, −0.02]**	**0.003**	**−0.15**
	Personal distress × stress	−0.01	[−0.03, 0.02]	0.73	−0.02
	**Perspective-taking × stress** ^**†**^	**0.03**	**[0.002, 0.06]**	**0.04**	**0.1**
	**Marginal *R*** ^**2**^ **/Conditional *R*** ^**2**^	0.3/0.65			

^†^*p* < 0.05, *p < 0.01, **p < 0.001, ***p < 0.0001. Significant effects are denoted in bold.

### Positive parenting

#### Empathic concern

Greater between-person empathic concern was marginally associated with higher positive parenting. Stress interacted with fluctuations in empathic concern. Further analysis (see Figure 3A) revealed that fluctuation in empathic concern was associated with positive parenting only for medium (*b* = 0.07, 95% CI [0.05, 0.1], *β* = 0.14, *p* = 6 × 10^−8^), and high (*b* = 0.14, 95% CI [0.09, 0.18], *β* = 0.29, *p* = 4 × 10^−8^) levels of stress. Fluctuations in empathic concern were not associated with positive parenting when stress levels were low (*b* = 0.01, 95% CI [−0.02, 0.04], *β* = 0.02, *p* = 0.44).

#### Perspective-taking

Fluctuations in perspective-taking throughout the lockdown were positively associated with positive parenting. This association was qualified by an interaction with stress (see Figure 3B) so that fluctuations in perspective-taking were associated with positive parenting only when stress levels were low (*b* = 0.06, 95% CI [0.03, 0.09], *β* = 0.11, *p* = 0.00001), while it was only nominally significant and did not pass multiple correction when stress levels were medium (*b* = 0.03, 95% CI [0.01, 0.06], *β* = 0.06, *p* = 0.01). However, fluctuations in perspective-taking were not associated with positive parenting when stress levels were high (*b* = 0.01, 95% CI [−0.03, 0.04], *β* = 0.02, *p* = 0.79).

### Parental accomplishment

#### Empathic concern

Like positive parenting, parental accomplishment was associated with individual differences in empathic concern. Moreover, similarly to the interaction found for positive parenting, fluctuations in empathic concern were found to interact with stress (see Figure 3C), with fluctuations in empathic concern associated with parental accomplishment only when stress levels were medium (*b* = 0.16, 95% CI [0.1, 0.22], *β* = 0.15, *p* = 0.0000003) or high (*b* = 0.27, 95% CI [0.17, 0.38], *β* = 0.23, *p* = 0.000001). Empathic concern was not associated with parental accomplishment when stress levels were low (*b* = 0.04, 95% CI [−0.03, 0.11], *β* = 0.03, *p* = 0.25).

#### Personal distress

Parental accomplishment was also negatively associated with both individual differences and fluctuations in personal distress.

#### Perspective-taking

Consistent with the results found for positive parenting, fluctuations in perspective-taking were also associated with parental accomplishment.

### Emotional exhaustion

#### Empathic concern

Fluctuations in empathic concern throughout the lockdown were positively associated with emotional exhaustion. However, this was qualified by interaction with stress. Further examination of this interaction (see Figure 3D) yielded marginally significant effects, with fluctuations in empathic concern positively associated with emotional exhaustion only when stress levels were low (*b* = 0.05, 95% CI [0.001, 0.09], *β* = 0.07, *p* = 0.047), while it was negatively associated with emotional exhaustion when stress levels were high (*b* = −0.09, 95% CI [−0.16, −0.02], *β* = −0.12, *p* = 0.01). No association between empathic concern fluctuations and emotional exhaustion was found in medium stress levels (*b* = −0.02, 95% CI [−0.06, 0.02], *β* = −0.02, *p* = 0.28).

#### Personal distress

Fluctuations in personal distress were positively associated with emotional exhaustion. Moreover, individual differences in personal distress were nominally associated with an increase in emotional exhaustion but did not pass multiple correction. The interaction between stress and individual differences in personal distress qualified this association (see Figure 3E) with a stronger association between personal distress and emotional exhaustion found as stress levels increased from low (*b* = 0.18, 95% CI [0.12, 0.24], *β* = 0.35, *p* = 4 × 10^−9^) to medium (*b* = 0.26, 95% CI [0.2, 0.31], *β* = 0.36, *p* = 2 × 10^−18^) to high (*b* = 0.33, 95% CI [0.26, 0.41], *β* = 0.39, *p* = 1 × 10^−17^).

#### Perspective-taking

Fluctuations in perspective-taking were negatively associated with parental emotional exhaustion. Although it did not pass multiple correction, fluctuation in perspective-taking nominally interacted with stress to predict emotional exhaustion (see Figure 3F), with changes in perspective-taking negatively associated with emotional exhaustion only when stress levels were low (*b* = −0.08, 95% CI [−0.13, −0.035], *β* = −0.125, *p* = 0.0005). This association was not found when stress levels were medium (*b* = −0.04, 95% CI [−0.07, 0.001], *β* = −0.05, *p* = 0.06), or high (*b* = 0.01, 95% CI [−0.06, 0.075], *β* = −0.01, *p* = 0.78).

## Discussion

This study aimed to examine how the different components of empathy interact with stress over time to predict key areas of psychosocial functioning: mental health, romantic relationships, and parenting. The results of this study are summarized in Table 5.

**Table 5:** Summary of results.

		Depressive symptoms	Relationship satisfaction	Positive parenting	Parental accomplishment	Emotional exhaustion
Individual differences	Empathic concern	–	–	Positive association^**†**^	Positive association	–
	Personal distress	Positive association increased as stress increased	Negative association increased as stress increased	–	Negative association	Positive association increased as stress increased
	Perspective taking	–	–	–	–	–
Fluctuations	Empathic concern	Negative association only when levels of stress are medium or high	–	Positive association only when levels of stress are medium or high	Positive association only when levels of stress are medium or high	Negative association only when levels of stress are medium or high
	Personal distress	Positive association increased as stress increased	Negative association with personal distress	–	Negative association	Positive association
	Perspective taking	Negative association only when stress levels were low^**†**^	–	Positive association only when stress levels were low	Positive association	Negative association only when stress levels were low^**†**^

^**†**^These results are nominally significant (*p* < 0.05 but not below the strict corrected *p* < 0.01 used in this study) and therefore should be interpreted with caution.

### Empathy association with psychosocial domains

Each empathy component showed unique patterns of associations with each psychosocial domain. The findings regarding depressive symptoms align with our hypothesis and previous research, which showed that depressive symptoms are positively related to personal distress and negatively related to cognitive empathy (Schreiter et al., 2013; Domes et al., 2016; Bennik et al., 2019). Our study adds to these findings by showing that in addition to personal distress, fluctuations in all empathic components, but not individual differences in these components, are associated with depressive symptoms under different stress levels.

In line with previous studies showing that relationship satisfaction strongly correlates with the emotional aspects of empathy (Davis and Oathout, 1987; Levesque et al., 2014; Righetti et al., 2016; Łada and Kaźmierczak, 2019), relationship satisfaction was only associated with personal distress. Personal distress was suggested to cause an ineffective reaction during emotional situations, such as engaging in more conflictual behaviors and/or showing less support for the partner, thus decreasing relationship satisfaction. Unlike these studies, we did not find positive associations between romantic satisfaction and other empathy components. The personal distress effect might have overshadowed the opposing but smaller effects of empathic concern and cognitive empathy, as self-focused (i.e., personal distress) and other-focused (i.e., empathic concern and perspective-taking) mechanisms can oppose each other (Łada and Kaźmierczak, 2019). This was true both for individual differences and fluctuations in personal distress.

Similar to previous studies, parenting was associated with all three components of empathy (Perez-Albeniz and de Paul, 2003, 2004; Soenens et al., 2007; Meidan and Uzefovsky, 2020). While all aspects of parenting in this study were related to fluctuations in cognitive empathy and empathic concern (under high levels of stress), specific patterns emerged for each aspect of parenting.

Parental exhaustion and parental accomplishment were related to individual differences in personal distress. Both aspects measure internal experiences aimed toward an appraisal of one’s parental functioning (see specific items in Supplementary Table S3). Personal distress is a self-focused reaction (Batson, 1991; Eisenberg et al., 2010). Therefore, we suggest that personal distress might directly cause or be the cause of transitions in internal experiences and perceptions of the parents regarding their parental functioning. This is in contrast with the positive parenting scale, which deals with more concrete parental behaviors directed toward the child. These results are also consistent when examining fluctuations in personal distress.

Additionally, parents with greater dispositional empathic concern reported higher parental accomplishment and positive parenting (although this was only nominally significant for the latter). This is consistent with previous findings suggesting empathic concern is related to child and parent-report of maternal warmth and caring behaviors (Soenens et al., 2007). As empathic concern evolved to facilitate caring behaviors toward one’s offspring (Stern and Cassidy, 2018), our findings provide another evidence for the relationship between empathic concern and parental behaviors and perceptions. Interestingly, only fluctuations, and not individual differences in cognitive empathy were also associated with these parental capacities.

### Empathy and stress interaction

Beyond specific differences between the domains assessed, each component of empathy consistently interacted with stress, supporting our hypothesis that stress might alter the way empathy relates to different psychosocial outcomes. Consistently, empathic concern was associated with outcomes, mainly during stressful times. We interpret this finding in light of Taylor’s evolutionary theory, which suggests that in times of stress, humans engage in tend-and-befriend responses, i.e., helping and caring behaviors that increase survival and fitness (Taylor et al., 2000; Taylor, 2011). Engaging in such behaviors may be proximally motivated by feelings of concern and care for others in distress—which is the definition of empathic concern (Wilhelm and Bekkers, 2010; Williams et al., 2014). Thus, fluctuations in empathic concern are specifically relevant to advantageous psychosocial outcomes during times of considerable stress, as the current findings show.

Alongside tend-and-befriend, stressful situations may also induce a self-focused personal distress response (Taylor, 2006; Decety and Lamm, 2011). We found that the associations between changes in the tendency to respond to others’ distress in a self-focused way were particularly relevant for negative outcomes under high stress conditions. Self-distress tends to lead to withdrawal and avoidance of the stressor (in this case, another’s distress) (Decety and Lamm, 2011). Therefore, it increases avoidance and inhibitory behaviors (Grynberg and López-Pérez, 2018; Eisenberg et al., 2019). Our data support that as stress increases, the feeling of personal distress becomes more demanding (and/or less manageable) and therefore more strongly relates to social avoidance behaviors. Such behaviors were previously found to increase depressive symptoms (Holahan et al., 2005), sabotage relationship satisfaction (Li and Chan, 2012), and increase parental exhaustion (Mikolajczak et al., 2018).

However, while the emotional components of empathy seem to be more strongly associated with outcomes under higher (or high) levels of stress, fluctuations in cognitive empathy were associated with three different outcomes only when stress levels were low (although two of them were only nominally significant). Unlike emotional empathy, which is a more automatic response, cognitive empathy is more effortful and cognitively demanding (de Waal and Preston, 2017). Under stress, automatic responses tend to become more dominant than controlled and effortful processes (Starcke and Brand, 2012; Hermans et al., 2014). Indeed, inducing stress indirectly increases prosocial behaviors by affecting emotional empathy, but not cognitive empathy (Tomova et al., 2016). Consistently, our findings suggest that stress modulates the effect of empathy on psychosocial outcomes, such that emotional empathy is more important in stressful situations, and cognitive empathy is more important in non-stressful situations, where considering the other’s perspectives, thoughts, and feelings becomes more valuable.

Taken together, the current findings emphasize the importance of fluctuations in empathy, showing that fluctuations in empathy (especially in empathic concern and cognitive empathy) are generally more predictive of personal and interpersonal constructs than individual differences in empathy. Furthermore, the interaction between empathy fluctuations and situational factors, such as stress, is highly predictive of psychosocial outcomes.

### Limitations

This study has several limitations. All measures used in our study were self-report questionnaires, which primarily reflect the participants’ perception of their functioning and ability. However, the current study focuses on participants’ self-perceptions and experiences, making self-report measures appropriate. Nevertheless, objective, implicit, and observational measures are needed to understand the mechanism underlying these effects. Furthermore, some measures such as the IRI (Davis, 1980) and parenting measures (Frick, 1991; Roskam et al., 2017) were adapted for weekly measures, and while these preserved high internal consistency, it might harm their validity to some extent.

Additionally, although the longitudinal design of this study offers a rich multilevel examination, this study is correlational, and causality cannot be conclusively determined based on its findings. While our findings support the “risky strength” formulation of empathy, whereby high levels of empathy can serve as a risk factor in some contexts (for review, see Tone and Tully, 2014), other paths linking empathy and different outcomes are also possible. For example, empathy might be viewed as the outcome and not as a predictor of the psychosocial aspects we measured. Future studies should examine the directionality between empathy and these outcomes.

Finally, this study was intentionally conducted during the first wave of the COVID-19 pandemic to investigate empathy ecologically in an ever-changing and stressful environment. COVID-19 imposed unique pressures (such as social isolation, home confinement, and/or health concerns) and therefore our findings may be limited to this era (for further discussion on this topic see Polizzi et al., 2020). Thus, further studies examining the relationships between trait and state empathy and psychosocial outcomes are needed.

## Conclusion

This study uniquely leverages the stressful and chaotic experience inflicted by the COVID-19 pandemic to examine the role of empathy in everyday life. Conducting intensive longitudinal research allowed us to simultaneously investigate how individual differences and fluctuations in multiple components of empathy interact with stress to predict major psychosocial domains of functioning.

This study has potential clinical and research implications. Our findings underscore the value of examining changes in empathy and its components showing they can serve as indicators of an individual’s psychosocial functioning and well-being. Moreover, the findings suggests that when designing interventions targeting empathy, it is crucial to consider the stress levels experienced by the individuals. In highly stressful circumstances, focusing on emotional components of empathy may be more effective, whereas targeting cognitive empathy may require a less stressful environment for achieving substantial changes in individuals’ lives.

Our study emphasizes the importance of considering each empathy component as these differently relate to psychosocial functioning and highlight the need to consider situational factors that could affect these links. Furthermore, the findings suggest that fluctuations in empathy might be more important than individual differences for preserving or deteriorating mental health, parental functioning, and romantic relationship outcomes. Consequently, our findings provide insights into the nature of human empathy and its relationships with key psychosocial outcomes.

## Data availability statement

The raw data supporting the conclusions of this article will be made available by the authors, without undue reservation.

## Ethics statement

The studies involving humans were approved by the Human Subject Committee of Ben-Gurion University. The studies were conducted in accordance with the local legislation and institutional requirements. The participants provided their written informed consent to participate in this study.

## Author contributions

IS designed the current study, analyzed the data, and wrote the manuscript. FU designed the current study and provided critical input on data analyses and the writing of the manuscript. AE provided critical comments on the manuscript and the writing of the manuscript. All authors read and approved the final version of the manuscript for submission.
