# Supplementary material for: Fluctuations and individual differences in empathy interact with stress to predict mental health, parenting, and relationship outcomes
Source: Front Psychol. 2023 Oct 19;14:1237278. doi: 10.3389/fpsyg.2023.1237278 (PMC10621795; doi:10.3389/fpsyg.2023.1237278)
Supplement: Supplementary file 1 [file Table_1.DOCX]

**Supplementary Information**


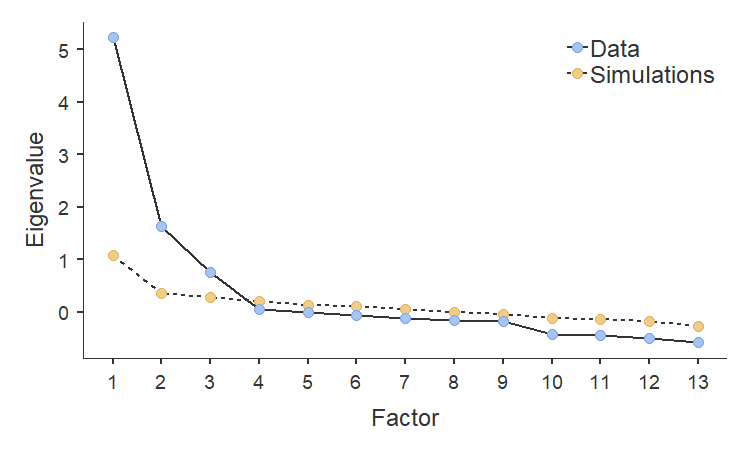


**Fig. S1.** Scree plot of the exploratory factor analysis of the parenting items. The solid line with blue points represents the eigenvalue of actual data. The dashed line with orange points shows the simulated data of the parallel analysis. According to this analysis, three factors should be retained as the eigenvalues in the actual data for these factors are higher than the simulated data.

**Table S1.** Summary of the number of parents and students that were excluded from each time point.

|  | Time point |  | 1 | 2 | 3 | 4 | 5 | 6 | 7 | 8 |
| --- | --- | --- | --- | --- | --- | --- | --- | --- | --- | --- |
|  | **Total number of participants** |  | **566** | **363** | **396** | **372** | **400** | **384** | **327** | **323** |
|  |  | Parents | 467 | 314 | 321 | 295 | 326 | 311 | 303 | 298 |
|  |  | Students | 99 | 49 | 75 | 77 | 74 | 73 | 24 | 25 |
| Exclusion criterion: | **Did not complete participation** |  | **12** | **2** | **8** | **7** | **3** | **6** | **3** | **6** |
|  |  | Parents | 12 | 2 | 8 | 7 | 2 | 5 | 3 | 5 |
|  |  | Students | 0 | 0 | 0 | 0 | 1 | 1 | 0 | 1 |
| Exclusion criterion: | **Did not pass attention check** |  | **8** | **3** | **9** | **8** | **17** | **9** | **12** | **4** |
|  |  | Parents | 2 | 2 | 6 | 5 | 13 | 3 | 11 | 2 |
|  |  | Students | 6 | 1 | 3 | 3 | 4 | 6 | 1 | 2 |
|  | **Number of participants after removal** |  | **546** | **358** | **379** | **357** | **380** | **369** | **312** | **313** |
|  |  | Parents | 453 | 310 | 307 | 283 | 311 | 303 | 289 | 291 |
|  |  | Students | 93 | 48 | 72 | 74 | 69 | 66 | 23 | 22 |

**Table S2.** Descriptive statistics of the sample.

| Time point | 1 | 2 | 3 | 4 | | 5 | 6 | 7 | 8 |
| --- | --- | --- | --- | --- | --- | --- | --- | --- | --- |
| Number of participants | 546 | 358 | 379 | | 357 | 380 | 369 | 312 | 313 |
| Sex (% females) | 77% | 75% | 76.50% | | 75% | 77% | 77% | 75% | 74% |
| Age | 31.8 (6.52) | 32.5 (6.35) | 32 (6.64) | | 31.8 (6.62) | 32.3 (6.54) | 32.2 (6.57) | 33.3 (6.1) | 33.3 (5.98) |
| Marital Status |  |  |  | |  |  |  |  |  |
| Single | 106 (19.4%) |  |  | |  |  |  |  |  |
| Married (or common-law partner) | 428 (78.4%) |  |  | |  |  |  |  |  |
| Divorced | 10 (1.8%) |  |  | |  |  |  |  |  |
| Widowed | 2 (0.4%) |  |  | |  |  |  |  |  |
| In a relationship | 88% |  |  | |  |  |  |  |  |
| Education |  |  |  | |  |  |  |  |  |
| Less than high school grad | 6 (1.1%) |  |  | |  |  |  |  |  |
| High school grad | 220 (40.3%) |  |  | |  |  |  |  |  |
| Bachelor's degree | 246 (45.05%) |  |  | |  |  |  |  |  |
| Master's/PhD | 74 (13.55%) |  |  | |  |  |  |  |  |
| Religion |  |  |  | |  |  |  |  |  |
| Jewish | 540 (99%) |  |  | |  |  |  |  |  |
| Muslim | 3 (0.5%) |  |  | |  |  |  |  |  |
| Other | 3 (0.5%) |  |  | |  |  |  |  |  |
| Income |  |  |  | |  |  |  |  |  |
| Much less than average | 66 (12.1%) |  |  | |  |  |  |  |  |
| Less than average | 85 (15.6%) |  |  | |  |  |  |  |  |
| Average | 189 (34.6%) |  |  | |  |  |  |  |  |
| More than average | 163 (29.8%) |  |  | |  |  |  |  |  |
| Much more than average | 31 (5.7%) |  |  | |  |  |  |  |  |
| Refused to answer | 12 (2.2%) |  |  | |  |  |  |  |  |
| Parenting status |  |  |  | |  |  |  |  |  |
| Parent | 447 (82%) |  |  | |  |  |  |  |  |
| Pregnant | 27 (5%) |  |  | |  |  |  |  |  |
| Median number of children for parents | 2±1  (min 1, max 11) |  |  | |  |  |  |  |  |
| Child's sex (% girls) | 47.60% |  |  | |  |  |  |  |  |
| Child's age in years | 5.02 ± 3.71 |  |  | |  |  |  |  |  |
| COVID-19 demographics |  |  |  | |  |  |  |  |  |
| At risk population | 49 (9%) |  |  | |  |  |  |  |  |
| Diagnosed with COVID-19 | 3 (0.5%) | 3 (0.84%) | 2 (0.53%) | | 0 (0%) | 3 (0.79%) | 1 (0.26%) | 1 (0.32%) | 3 (0.96%) |

| **Table S3.** Exploratory factor analysis of the parenting items | | | | | | | | | | | | | | | | | |
| --- | --- | --- | --- | --- | --- | --- | --- | --- | --- | --- | --- | --- | --- | --- | --- | --- | --- |
|  | | **Factor** | | | | | | | | | | | |  |  |  |  |
|  | | **1** | | | | **2** | | | | **3** | | | |  |  |  |  |
| I am at the end of my patience at the end of a day with my children (EE) |  |  | |  | |  | |  | | 0.619 | |  | |  | |  |  |
| being a parent every day requires a good deal of effort (EE) |  |  | |  | |  | |  | | 0.706 | |  | |  | |  |  |
| It stresses me too much to take care of my children (EE) |  |  | |  | |  | |  | | 0.738 | |  | |  | |  |  |
| I feel like my parental role is breaking me down (EE) |  |  | |  | |  | |  | | 0.830 | |  | |  | |  |  |
| I aim easily able to understand what my children feel (PA) |  | 0.524 | |  | |  | |  | |  | |  | |  | |  |  |
| I look after my children’s problems very effectively (PA) |  | 0.754 | |  | |  | |  | |  | |  | |  | |  |  |
| Through my parental role, I feel that I have a positive influence on my children (PA) |  | | 0.925 | |  | |  | |  | |  | |  | |  | |  |
| I am easily able to create a relaxed atmosphere with my children (PA) |  | 0.717 | |  | |  | |  | |  | |  | |  | |  |  |
| I accomplish many worthwhile things as a parent (PA) |  | 0.777 | |  | |  | |  | |  | |  | |  | |  |  |
| As a parent, I handle emotional problems very calmly (PA) |  | 0.767 | |  | |  | |  | |  | |  | |  | |  |  |
| You let your child know when he/she is doing a good job with something (PP) |  |  | |  | | 0.850 | |  | |  | |  | |  | |  |  |
| You compliment your child after he/she has done something well (PP) |  |  | |  | | 0.972 | |  | |  | |  | |  | |  |  |
| You praise your child if he/she behaves well (PP) |  |  | |  | | 0.911 | |  | |  | |  | |  | |  |  |
|  | | | | | | | | | | | | | | | | | |

 Factor loadings of the exploratory factor analysis of the parenting items. Three factors were optimally chosen, replicating the original structure of the three subscales used. EE – emotional exhaustion; PA – parental accomplishment; PP – positive parenting.

**Table S4.** List of models used in the multilevel modeling analysis (MLM).

| Model | Specification |
| --- | --- |
| Model 1 | null model |
| Model 2 | Model 1 + sex and age |
| Model 3 | Model 2 + random intercept of the outcome |
| Model 4 | Model 3 + random and fixed effect of empathic concern, personal distress and perspective-taking (empathy components) |
| Model 5 | Model 4 + Stress |
| Model 6 | Model 5 + Stress and empathy components interaction |

MLM analyses were conducted separately for depressive symptoms, positive parenting, parental accomplishment, emotional exhaustion, and relationship satisfaction.

|  | Time point | 1 | 2 | 3 | 4 | 5 | 6 | 7 | 8 |
| --- | --- | --- | --- | --- | --- | --- | --- | --- | --- |
| Empathy | Empathic concern | 20.8 (3.78) | 20.6 (3.65) | 20.2 (4.09) | 20.3 (3.84) | 19.9  (4) | 19.8 (4.41) | 19.7 (4.35) | 19.8 (4.12) |
|  | Personal distress | 12.6 (3.8) | 12.1 (3.79) | 12 (3.79) | 11.7 (4.15) | 11.6 (4.12) | 11.1 (4.23) | 10.9 (4.15) | 11 (4.15) |
|  | Perspective-taking | 17.9 (4.04) | 18.1 (3.96) | 17.6 (4.41) | 18 (4.74) | 18 (4.66) | 17.9 (4.8) | 18 (4.77) | 18.1 (4.89) |
| Depression |  | 3.31 (2.96) | 2.93 (2.89) | 3.44 (3.35) | 3.21 (3.1) | 3.19 (3.25) | 2.7 (3.05) | 2.51 (3.37) | 2.25 (3.1) |
| Stress |  | 2.74 (1.12) | 2.43 (1.09) | 2.42 (1.11) | 2.42 (1.11) | 2.38 (1.12) | 2.29 (1.1) | 2.2 (1.16) | 2.18 (1.12) |
| Parenting | Positive parenting | 11.2 (1.4) | 10.7 (1.8) | 10.9 (1.68) | 11 (1.74) | 10.9 (1.76) | 11 (1.73) | 11 (1.89) | 11.1 (1.7) |
|  | Parental accomplishment | 19.1 (3.37) | 18.9 (3.44) | 18.7 (3.66) | 19.2 (3.89) | 19.1 (4.O1) | 19.6 (3.86) | 20.1 (4.26) | 20.1 (3.87) |
|  | Emotional exhaustion | 8.09 (2.51) | 7.73 (2.61) | 7.77 (2.76) | 7.52 (2.77) | 7.3 (2.84) | 7.03 (2.69) | 6.4 (2.62) | 6.25 (2.42) |
| Relationship satisfaction |  | 19.8 (4.69) | 19 (5.12) | 18.8 (5.31) | 19.2 (5.2) | 19 (5.15) | 19.3 (4.99) | 19.1 (5.38) | 19.6 (4.94) |

**Table S5**. Descriptive statistics of the main variables and outcomes across the 8 different time points.
